# Nilotinib Improves Bioenergetic Profiling in Brain Astroglia in the 3xTg Mouse Model of Alzheimer’s Disease

**DOI:** 10.14336/AD.2020.0910

**Published:** 2021-04-01

**Authors:** Aida Adlimoghaddam, Gary G Odero, Gordon Glazner, R. Scott Turner, Benedict C Albensi

**Affiliations:** ^1^Division of Neurodegenerative Disorders, St. Boniface Hospital Research, Winnipeg, MB, Canada.; ^2^Department of Pharmacology & Therapeutics, University of Manitoba, Winnipeg, MB, Canada.; ^3^Department of Neurology, Georgetown University, Washington, DC, USA.

**Keywords:** mitochondrial function, bioenergetics, biogenesis, Alzheimer’s disease, astroglia, nuclear factor kappa B (NF-κB), cytochrome c oxidase, citrate synthase, oxidative phosphorylation

## Abstract

Current treatments targeting amyloid beta in Alzheimer’s disease (AD) have minimal efficacy, which results in a huge unmet medical need worldwide. Accumulating data suggest that brain mitochondrial dysfunction play a critical role in AD pathogenesis. Targeting cellular mechanisms associated with mitochondrial dysfunction in AD create a novel approach for drug development. This study investigated the effects of nilotinib, as a selective tyrosine kinase inhibitor, in astroglia derived from 3xTg-AD mice versus their C57BL/6-controls. Parameters included oxygen consumption rates (OCR), ATP, cytochrome c oxidase (COX), citrate synthase (CS) activity, alterations in oxidative phosphorylation (OXPHOS), nuclear factor kappa B (NF-κB), key regulators of mitochondrial dynamics (mitofusin (Mfn1), dynamin-related protein 1 (Drp1)), and mitochondrial biogenesis (peroxisome proliferator-activated receptor gamma coactivator1-alpha (PGC-1α), calcium/calmodulin-dependent protein kinase II (CaMKII), and nuclear factor (erythroid-derived 2)-like 2 (Nrf2)). Nilotinib increased OCR, ATP, COX, Mfn1, and OXPHOS levels in 3xTg astroglia. No significant differences were detected in levels of Drp1 protein and CS activity. Nilotinib enhanced mitochondrial numbers, potentially through a CaMKII-PGC1α-Nrf2 pathway in 3xTg astroglia. Additionally, nilotinib-induced OCR increases were reduced in the presence of the NF-κB inhibitor, Bay11-7082. The data suggest that NF-κB signaling is intimately involved in nilotinib-induced changes in bioenergetics in 3xTg brain astroglia. Nilotinib increased translocation of the NF-κB p50 subunit into the nucleus of 3xTg astroglia that correlates with an increased expression and activation of NF-κB. The current findings support a role for nilotinib in improving mitochondrial function and suggest that astroglia may be a key therapeutic target in treating AD.

Alzheimer’s disease (AD) is a multifactorial neurodegenerative disorder, characterized by progressive cognitive impairments [1]. Approaches focusing on therapeutics that interfere with amyloid β plaques (Aβ), a classic hallmark of AD [2, 3], have failed for the most part to show efficacy for stopping or slowing cognitive decline. However, accumulating evidence demonstrates mitochondrial dysfunction early in the pathology of human diseases, cardiovascular, neurodegenerative, and cancers, which may serve as an important and primary therapeutic target [4-9]. Low doses of the anti-cancer drug nilotinib suggest cognitive benefits for patients with Parkinson's disease (PD) and related dementias [10, 11]. Nilotinib (Tasigna^®^, AMN107) is a c-Abl tyrosine kinase inhibitor approved by the Food and Drug Administration (FDA) for patients with chronic myeloid leukemia (CML) [12, 13]. Compared to the other c-Abl inhibitors, low dose nilotinib is more advantageous for penetrating the blood brain barrier (BBB) and degrades α-synuclein through a process of autophagy in PD models [14-17]. Safety and efficacy of nilotinib in AD patients has been tested. It has recently been reported that biomarkers of AD were changed as a function of nilotinib treatment [18]. Moreover, nilotinib enhances dopaminergic neurons and improves motor function in PD and Lewy body dementia (LBD) [10, 14]. The level of c-Abl is elevated in post-mortem brains of patients with AD and PD [19-21]. Proteomic analyses show that c-Abl phosphorylates tau tangles in AD brains [19]. In PD models, c-Abl increased oxidative stress, presumably due to the accumulation of dysfunctional mitochondria that generate and release reactive oxygen species (ROS) [21, 22]. Overexpression of glial c-Abl leads to inflammation and neurodegeneration [23, 24]. Under oxidative stress conditions, c-Abl is activated in astroglia and blocking c-Abl prevented the oxidative death of astroglia [25]. More research identified that overexpression of glial c-Abl contributes to both oxidative stress as well as inflammation in a mouse pain model [24]. Moreover, c-Abl inhibitors reduced both inflammatory activation of glia and chronic pain [26].

Astroglia, as important central nervous system (CNS) cells, play key roles in the pathogenesis of neurodegenerative disorders such as AD; excessive oxidative stress, inflammation as well as mitochondrial impairment is observed in AD. Accumulating evidence highlights the critical role of astroglia in inflammatory, oxidative stress, modulation of synaptic activity, and bioenergetic changes present in AD [27-31]. According to all of these functional properties, astroglia have become exciting targets for our study. The effect of nilotinib on metabolic activity and inflammation in astroglia, however, has not been examined.

The notion of testing nilotinib for AD may seem counterintuitive since AD involves cell loss and cancer involves unregulated cell proliferation, *but* the earliest deficits in the pathological progression of AD *and* cancer are associated with mitochondrial dysfunction; that is, before the robust appearance of neurotoxic proteins in AD and oncogene expression in cancer [4-8, 32-39].

Therefore, targeting mitochondrial bioenergetics may be a promising new strategy for treating AD [5, 40-44]. Interestingly, mitochondrial function is considered as a target for cancer therapy, but has not been exploited for AD. Reprograming metabolic activity of cancer cells led to non-apoptotic function [45]. This finding indicates that apoptotic pathway is linked to mitochondria and metabolic pathways. Therefore, the linkage between cancer and AD provides a rational for repurposing existing anticancer drugs for AD.

Previously, our laboratory documented reductions in brain metabolic activity and in the expression of mitochondrial protein subunits (Complexes I-V) in 3xTg brain samples estimated to compromise the site of oxidative phosphorylation (OXPHOS) and mitochondrial efficiency through the action of ATP synthesis [8]. Beside down-regulation of OXPHOS capacity in AD, reduced activity of other mitochondrial metabolic enzymes such as citrate synthase (CS) is associated with AD [46]. Both mitochondrial dynamics (fission and fusion) and number are significantly altered in AD models that ultimately results in mitochondrial dysfunction [47-50]. The effects of nilotinib on mitochondrial function, however, have not been examined. The regulation of mitochondrial function is also linked to nuclear factor kappa B (NF-κB) signaling, a highly-conserved pathway for immune function, mitochondrial function and previously studied in AD *and* cancer [43, 51-53]. Some studies reported that NF-κB signaling regulates Aβ-induced mitochondrial function and cytochrome c oxidase (COX) activity [54, 55]. Other studies confirm that the enzymatic activity of COX may be modulated by the NF-κB signaling pathway [54, 56]. NF-κB plays a crucial role in the metabolic activity of AD *and* cancer [53,57]. Several studies have also shown that NF-κB activity restricts activation of inflammasomes through the removal of damaged mitochondria [53, 58-60]. Whether the mitochondrial-related NF-κB signaling pathway is affected by nilotinib is not known.

Mitochondrial function is partially regulated by calcium/calmodulin-dependent protein kinase (CaMK), a downstream target of the NF-κB, signaling pathway [61]. CaMK has been shown to be a key protein in neurodegeneration, metabolic regulations, and biogenesis [62]. Peroxisome proliferator-activated receptor gamma coactivator 1 (PGC-1α), a downstream target of CaMK, has been implicated in mitochondrial biogenesis [63]. Further, PGC-1α activates transcription factor nuclear factors (Nrf) and mitochondrial transcription factor A (TFAM), known to regulate mitochondrial biogenesis [64, 65]. The effect of nilotinib on mitochondrial biogenesis in astroglia, however, has not been investigated.

**Figure 1.**
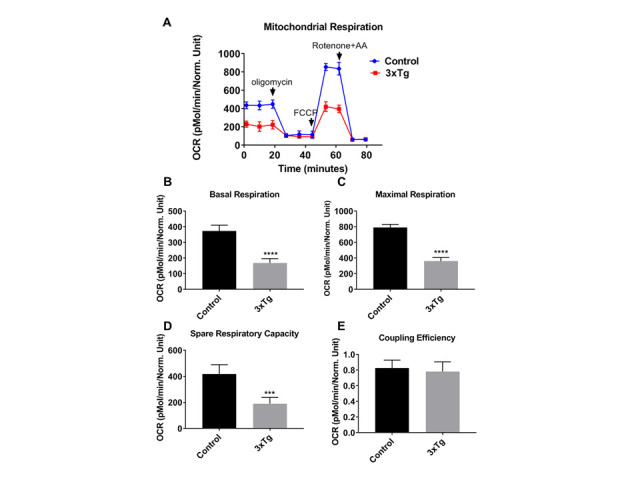
Mitochondrial respiration rates are reduced in 3xTg-AD astroglia. (A) Kinetics graph indicating real-time OCR at baseline and after addition of oligomycin, FCCP, and rotenone/antimycin. The OCR was measured in 3xTg and C57BL/6-WT astroglia utilizing the XF24 analyzer. (B) Basal respiration, (C) maximal respiration, (D) spare respiratory capacity, and (E) Coupling efficiency were calculated compared between 3xTg and control cells. Data are mean ± SD of n = 6 per group (*** = p < 0.001 or **** = p < 0.0001) analyzed by unpaired Student's t-test.

The aim of this study was to test the hypothesis that nilotinib improves astroglia mitochondrial function. We performed bioenergetic profiling in AD *vs*. wild type (WT)/control astroglia while in the presence and absence of nilotinib. Specifically, we measured 1) oxygen consumption rates (OCR) and ATP levels; 2) enzymatic activity of COX and CS; 3) levels of OXPHOS; 4) numbers of mitochondria; 5) levels of proteins involved in mitochondrial dynamics (e.g., Mfn1, Drp1); 6) levels of proteins involved in mitochondrial biogenesis (e.g., CaMKII, PGC-1α, Nrf2, and TFAM); 7) translocation, activation, and expression levels of NF-κB subunit proteins (NF-κB p50, p105, p65, and p75) and IκB-α; and 8) effects of NF-κB inhibition on cellular respiration and ATP levels.

**Figure 2.**
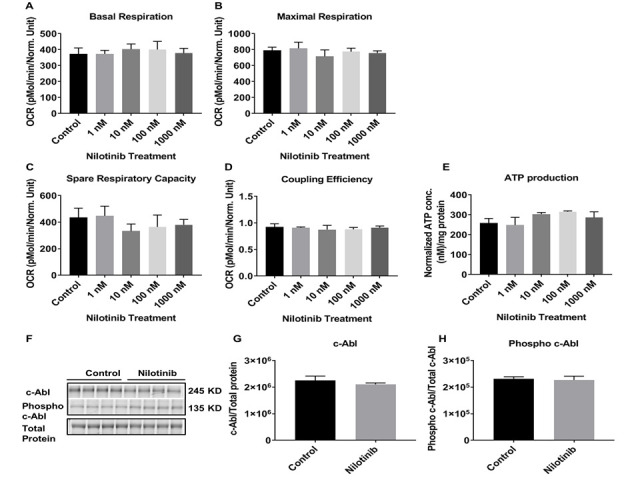
Nilotinib did not alter mitochondrial respiration rate or expression of total or phospho c-Abl in C57BL/6-WT astroglia. The OCR was measured in C57BL/6 astroglia utilizing the XF24 analyzer after 24 hours treatment with nilotinib (1, 10, 100, and 1000 nM). (A) Basal respiration, (B) maximal respiration, (C) spare respiratory capacity, (D) Coupling efficiency, and (E) total ATP level were calculated and compared between control and treated cells. Western blot experiments demonstrating relative levels of c-Abl and p-c-Abl in C57BL/6-WT astroglia (F) in the presence and absence of 24 hrs. 100 nM nilotinib treatment. Relative quantification for protein levels of c-Abl and p-c-Abl normalized to total protein and total c-Abl, respectively (G, H). Data are mean ± SD of n = 6 per group (* = p < 0.05) analyzed by unpaired Student's t-test.

## MATERIALS AND METHODS

### Animals

We utilized both female and male pups (5-7 days) from 3xTg mice. This transgenic model of AD expresses hAPP_Swe_, hPS1_M146V_, and hTau_P301L_ mutations, and exhibit synaptic dysfunction. As a control, female and male pups (5-7 days) derived from wild-type C57BL/6 mice were used. Mice were given *ad libitum* access to food and water and housed under the standard light*/*dark cycle (12 h light, 12 h dark at room temperature (22°C)). All animal procedures followed guidelines of the University of Manitoba Animal Care Committee, the Canadian Council of Animal Care rules, and the Institutional Animal Care and Use Committee (IACUC) standards.

**Figure 3.**
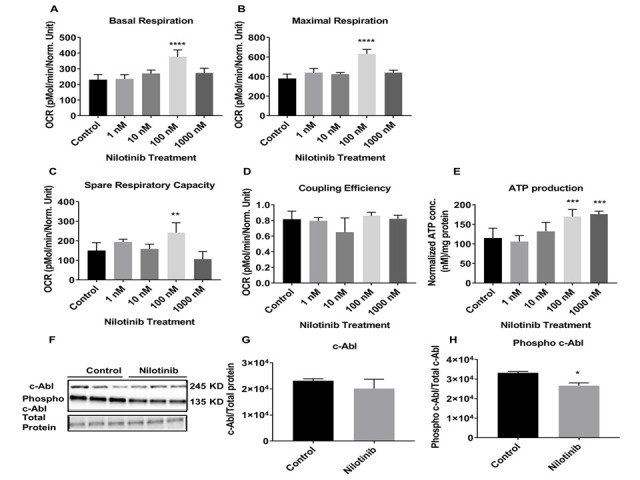
Nilotinib improved mitochondrial respiration rates in 3xTg-AD astroglia. Nilotinib inhibited the expression level of phospho c-Abl in 3xTg astroglia. The OCR was measured in 3xTg-AD astroglia utilizing the XF24 analyzer after 24 hours treatment with nilotinib (1, 10, 100, and 1000 nM). (A) Basal respiration, (B) maximal respiration, (C) spare respiratory capacity, (D) coupling efficiency, and (E) total ATP level were calculated and compared between control and treated cells. Western blot experiments demonstrating relative levels of c-Abl and p-c-Abl in 3xTg-AD astroglia (F) in the presence and absence of 24 hrs. 100 nM nilotinib treatment. Relative quantification for protein levels of c-Abl and p-c-Abl normalized to total protein and total c-Abl, respectively (G, H). Data are mean ± SD of n = 6 per group (* = p < 0.05 or ** = p < 0.01 or *** = p < 0.001 or **** = p < 0.0001) analyzed by unpaired Student's t-test.

### Primary cultures of astroglia

Primary cortical astroglia derived from both AD (3xTg) and control (C57BL/6) pups (5-7 days) were prepared as previously described [66]. Briefly, cortical tissue was dissected and placed into a chilled dissection medium [67] composed of Ca^2+^ and Mg^2+^- free Hanks’ balanced salt solution (HBSS), pH 7.4, containing the following: 137 mM NaCl, 5.36 mM KCl, 0.27 mM Na_2_HPO_4_, 1.1 mM KH_2_PO_4_, and 6.1 mM glucose. Isolated cortical tissue was sliced using a razor. Following slicing, tissues were gently separated from each other in fresh cold dissecting media and mechanically dissociated by sequential passage through a Pasteur pipette. After dissociation, we centrifuged the cells at 1,000 rpm for 5 min, and the pellet was re-suspended in Dulbecco’s modified Eagle’s medium (DMEM)/F12 culture medium (Gibco, Grand Island, NY, USA) supplemented with 10% fetal bovine serum (FBS), 0.5 mg mL^-1^ streptomycin, 0.5 U mL^-1^ penicillin (Invitrogen, Carlsbad, CA, USA), 8.39 mM HEPES, and 23.8 mM NaHCO_3_. Cells derived from 3xTg-AD and C57Bl/6 pups were plated on 25-cm^2^ flasks at a density of 6×10^5^ cells cm^-2^ and grown at 37 °C using an incubator with humidified 5% CO_2_ and 95% air. After 12 days, the cell culture reached confluence. Cultures were then shaken gently, and any microglia/floating cells were removed, [68, 69] resulting in more than 90% pure culture of astroglial, as described previously (Supplementary Fig. 1).

**Figure 4.**
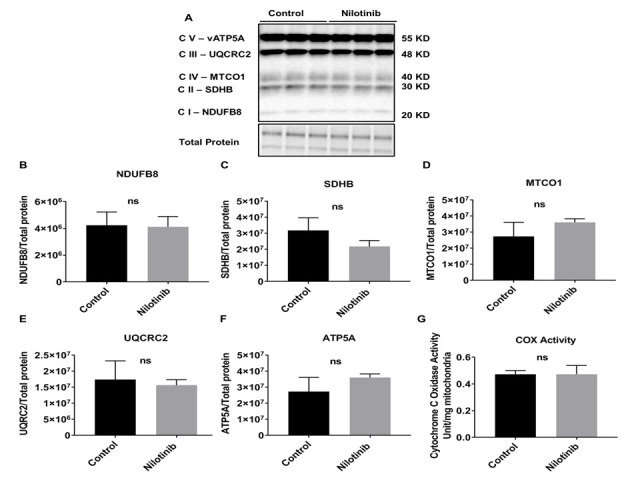
Nilotinib did not alter the expression of mitochondrial Complex (I-V) protein subunits in C57BL/6-WT astroglia. Western blot experiments demonstrating relative levels of mitochondrial protein subunits in C57BL/6-WT astroglia in the presence and absence of 24 hrs. 100 nM nilotinib treatment. (A) Representative Western blot for NADH dehydrogenase beta sub complex subunit 8 of Complex I (NDUFB8), succinate dehydrogenase subunit B of Complex II (SDHB), cytochrome b-c1 complex subunit 2 of Complex III (UQCRC2), Cytochrome c oxidase subunit 1 of Complex IV (MTCO1), and ATP synthase subunit alpha of Complex V (ATP5A). (B-F) Relative quantification for protein levels of Complex I-V normalized to total protein. (G) COX activity was measured in C57BL/6-WT astroglia in presence and absence of 100 nM nilotinib treatment. Results are expressed as mean ± SD of n = 6 per group (*P ≤ 0.05) analyzed by unpaired Student's t-test.

**Figure 5.**
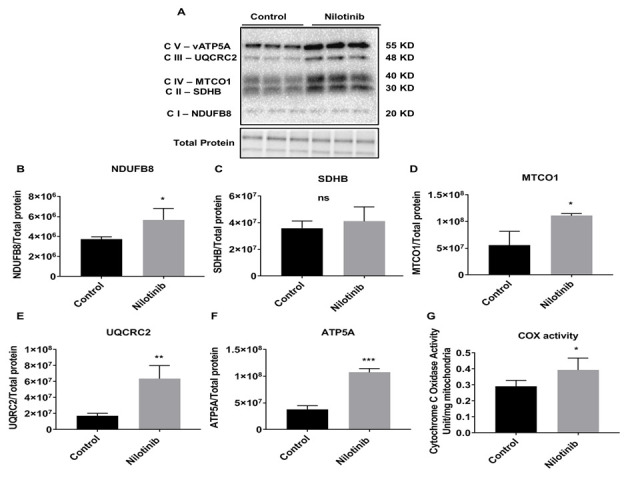
Nilotinib significantly increased the expression of mitochondrial Complex (I and III-V) protein subunits and cytochrome c oxidase (COX) activity in 3xTg-AD astroglia. Western blot experiments demonstrating relative levels of mitochondrial protein subunits in 3xTg astroglia in the presence and absence of 24 hrs. 100 nM nilotinib treatment. (A) Representative Western blot for NADH dehydrogenase beta subcomplex subunit 8 of Complex I (NDUFB8), succinate dehydrogenase subunit B of Complex II (SDHB), cytochrome b-c1 complex subunit 2 of Complex III (UQCRC2), Cytochrome c oxidase subunit 1 of Complex IV (MTCO1), and ATP synthase subunit alpha of Complex V (ATP5A). (B-F) Relative quantification for protein levels of Complex I-V normalized to total protein. (G) COX activity was measured in 3xTg-AD astroglia in presence and absence of 100 nM nilotinib treatment. Results are expressed as mean ± SD of n = 6 per group (*P ≤ 0.05**P ≤ 0.01) analyzed by unpaired Student's t-test.

### Measurement of mitochondrial respiration rates

Astroglia derived from 3xTg (AD) and C57BL/6 (control) mice were cultured separately on Seahorse XF24 plates (Seahorse Biosciences, Billerica, MA). To investigate whether nilotinib increased mitochondrial respiration in 3xTg astroglia through the NF-κB signaling pathway, astroglia were treated with either Bay11-7082, a well-known NF-κB inhibitor that has pharmacological activities including neuroprotective, anticancer, and anti-inflammatory roles [70], or co-treated with nilotinib and Bay11-7082 for 24 hours. Following treatment, cells were subjected to un-buffered DMEM supplemented with 1 mM sodium pyruvate, 1 mM glucose, at pH 7.4 and incubated in a non- CO_2_ incubator for one hour at 37 °C. First, the basal oxygen consumption rate was measured before injection of selected mitochondrial inhibitors. Second, oligomycin (1 µM), an irreversible ATP synthase inhibitor, was added to drive a proton gradient. Third, carbonyl cyanide-p-trifluoromethoxyphenyl-hydrazone, a.k.a FCCP (1 µM), an electron transport chain (ETC) uncoupler, was added to lower the proton gradient and allow for maximum respiration of cells. Fourth, rotenone (1 µM), a Complex I inhibitor, and antimycin A (1 µM), a Complex III inhibitor, were added to cease respiration, thus blocking mitochondrial electron transfer at cytochrome c oxidase. Following the addition of rotenone and antimycin, basal respiratory data were calculated and corrected with subtraction of non-mitochondrial respiration rates. Maximal respiration rate was calculated by dividing FCCP-stimulated OCR by OCR oligomycin-stimulated OCR. Spare respiratory capacity (ability of the cell to respond to improved energy demand) was approximated by measuring the differences between basal and maximal respiration rates. Coupling efficiency is the proportion of OCR that is utilized to make ATP and was estimated as the fraction of basal mitochondrial OCR used for ATP synthesis (ATP-linked OCR/basal OCR).

For analysis, OCR readings were measured and recorded by Seahorse XF-24 software (Seahorse Biosciences, Billerica, MA). Finally, the OCR level was normalized based on protein concentrations colorometric detergent compatible (DC) protein assay kit (BioRad, Hercules, California, USA)) for each well. The OCR measures from each well were normalized to total protein levels.

### Measurement of cellular ATP

ATP production was determined with a luminescent ATP detection assay kit (ab113849: Abcam, Cambridge, UK) as per manufacturer's instructions. Briefly, cells were washed with phosphate buffered saline (PBS). Then, ATP standard dilutions were made. Further, cells and ATP standard dilutions were incubated with detergent at room temperature (RT) for five minutes on an orbital shaker. After 10 minutes incubation in the dark, ATP levels were measured on a Micoplate Reader (Dynex Technologies, Denkendorf, Germany) by tracking the change of absorbance at 535/587nm.

### Measurement of cytochrome c oxidase and citrate synthase enzymatic activity

The activity of cytochrome c oxidase (COX) and citrate synthase (CS) was assessed spectrophotometrically (*Ultrospec 2100 pro;* GE Healthcare) using *ScienCell* assay kits (8278 and 8318, *ScienCell* Research Laboratory, California, USA) on isolated mitochondria derived from control and 3xTg astroglia. The activity of COX and CS was determined spectrophotometrically by measuring absorbance at 550 nm, 412 nm, respectively.

All relative quantification for enzyme assays were analyzed based on densitometry values normalized to total protein level. Protein concentrations were measured utilizing a colorometric DC protein assay kit (BioRad, Hercules, California, USA).

### Nuclear protein extraction and electrophoretic mobility shift assay (EMSA)

Nuclear protein was isolated from astroglia cultures using the NE-PER™ nuclear and cytoplasmic extraction kit (ThermoFisher Scientific, San Jose, CA, USA) as per the manufacturer’s instructions. EMSA, used to study protein: DNA interactions, was performed using the LightShift™ Chemiluminescent EMSA Kit (ThermoFisher Scientific, San Jose, CA, USA). For the EMSA, 25 μL reaction mixtures containing binding buffer (10x binding buffer, 0.05μg/μL Poly (dI•dC), 2.5% glycerol, 0.05% NP40, 5mM MgCl2, 50mM KCl, 1mM EDTA, 0.5mg/mL BSA), 15 μg nuclear protein (astroglia) and 40 fM biotinylated probe and 20 fM biotinylated probe, respectively, corresponding to a NF-κB-binding site (5′-AGTTGAGGGGACTTTCCCAGGC-3′) were incubated at room temperature for 30 minutes. For the competition reaction, 100 pM unbiotinylated probe was added to the nuclear protein/binding buffer reaction for 20 minutes at room temperature after which a 40 fM biotinylated probe was added for 30 minutes at room temperature. A total of 20 μL of reaction mixture was loaded on native 6% polyacrylamide gels prepared in 0.5× TBE and electrophoresed at 100V for 90 minutes at room temperature. Gels were then transferred onto a nylon membrane for 90 minutes at 4°C at 380mA then UV cross-linked for one minute at 120 mJ/cm^2^. The processing of the membrane was performed using the Chemiluminescent Nucleic Acid Detection Module Kit (ThermoFisher Scientific, San Jose, CA, USA) as per the manufacturer’s instructions. The nylon membrane was imaged with the Chemidoc™ MP (Bio-Rad, Hercules, CA, USA).

### Protein extraction and Western blot analysis

Proteins were stored at -80 °C prior to Western blotting as previously described [8]. Briefly, astroglia were harvested from the cortex of C57BL/6-control and 3xTg-AD mice and then homogenized in ice-cold radioimmuno-precipitation (RIPA) buffer containing: 150 mM sodium chloride, 50 mM Tris, pH 8.0, 0.5% sodium deoxycholate, 0.1% sodium dodecyl sulfate (SDS), 1% Triton X-100, 1% phosphatase inhibitor cocktail (Sigma-Aldrich, St. Louis, MO, USA), and 1% protease inhibitor cocktail (Amresco, Solon, OH, USA). Further, protein concentrations were quantified utilizing a colorometric DC protein assay kit (BioRad, Hercules, California, USA). Further, a 4X Laemmli buffer (20% β-mercaptoethanol, 16% SDS, 40% glycerol, 0.01% bromophenol blue, and 0.25M Tris, pH 6.8) was added to the samples, followed by denaturing at 50˚C for 8 minutes. Equal amounts of proteins (15 ug per well) were loaded in each well of a 10% polyacrylamide SDS-PAGE gels (Bio-Rad, Hercules, CA, USA) and electrophoresed with a Tris-glycine running buffer at 200 volts for 45 minutes. Gels were then activated using a ChemiDoc™ MP imager (Bio-Rad, Hercules, CA, USA) and transferred to a 0.2 µm nitrocellulose membranes (Bio-Rad Hercules, CA, USA) with the Trans-Blot® Turbo™ Transfer System (Bio-Rad, Hercules, CA, USA) and total protein on the membranes were measured utilizing the ChemiDoc™ MP imager. Following imaging, all membranes were blocked in Tris-buffered saline with 0.1% Tween-20 (TBS-T) with 5% milk, with the exception of membranes detecting phosphorylated proteins, which were blocked in TBS-T with 5% bovine serum albumin (BSA) instead of 5% milk, for one hour on a tilting platform at room temperature. Following blocking, membranes were then incubated at 4°C overnight with the following primary antibodies: Total OXPHOS Rodent WB Antibody Cocktail (ab110413, Abcam, Cambridge, UK; 1:1000), NF-κB p105/p50 (ab32360, Abcam, Cambridge, UK; 1:1000), NF-κB p65 (ab16502, Abcam Cambridge, UK; 1:1000), NF-κB p75 (108299, Abcam, Cambridge, UK; 1:1000), IκB-α (ab32518, Abcam, Cambridge, UK; 1:500), Drp1 (ab156951, Abcam, Cambridge, UK; 1:200), Mfn1 (ab56889, Abcam, Cambridge, UK; 1:500), c-Abl (2862, Cell signaling technology, Danvers, USA; 1:100), phospho-c-Abl (Tyr245) (2868, Cell signaling technology, Danvers, USA; 1:100), PGC-1α (PA5-38022, Thermo Fisher Scientific, Massachusetts, USA; 1:500), CaMKII (M-176, Santa Cruz, Texas, USA; 1:500), Nrf2 (ab62352, Abcam, Cambridge, UK; 1:500), and TFAM (ab131607, Abcam, Cambridge, UK; 1:500). Following primary antibody incubation, all membranes were washed with TBS-T buffer (three times; fifteen minutes each) and then incubated with either goat anti-mouse, or anti-rabbit IgG (H+L) antibody (Jackson ImmunoResearch Laboratories, West Grove, PA, USA, 1:2000 dilution) prepared in TBS-T buffer with 5% milk or BSA for one hour at 4 °C. Later, membranes were washed with 1X TBS-T buffer (three times; fifteen minutes each) and subjected to enhanced chemiluminescence ECL, utilizing a clarity ECL kit (Bio-Rad, Hercules, CA, USA) for five minutes and then visualized by the ChemiDoc™ MP imager using Bio-Rad Image Lab software.

**Figure 6.**
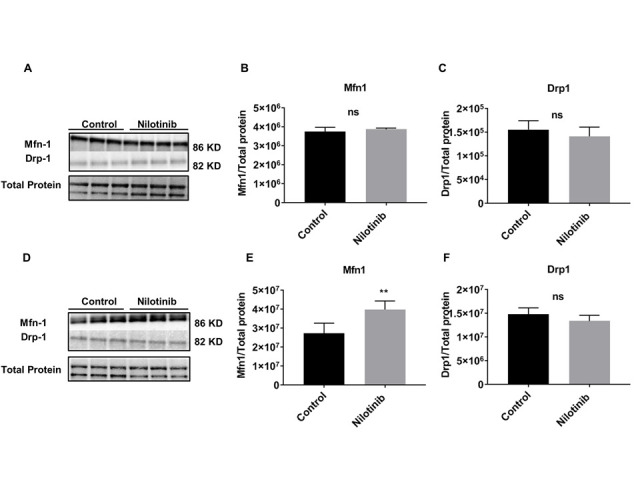
Nilotinib significantly altered the expression of Mfn1 in 3xTg-AD astroglia. Western blot experiments demonstrating relative levels of Mfn1 and Drp1 in C57BL/6-WT astroglia (A-C) and 3xTg astroglia (D-F) inthe presence and absence of 24 hrs. 100 nM nilotinib treatment. Relative quantification for protein levels of Mfn-1 and Drp-1 normalized to total protein. Results are expressed as mean ± SD of n = 6 per group (***P ≤ 0.001) analyzed by unpaired Student's t-test.

### Immunohistochemistry

Immunohistochemistry was utilized to detect astroglia as described previously [8]. Briefly, cells were rehydrated using 1X TBS with 0.1% Tween 20 (TBS-T), for five minutes. Following rehydration, astroglia were fixed with 4% paraformaldehyde in PBS (pH 7.4) for 10 min at room temperature. Further, antigen retrieval was performed for 20-30 minutes in preheated (80 °C) citrate buffer (PH 8). All cells were then washed tree times with TBS-T (each time: 5 minutes each). Further, cells were then treated with anti-glial fibrillary acidic protein (GFAP) antibody (ab16997, abcam Cambridge, Massachusetts, USA, 1:100 dilution) to detect astrocytes combined with the EXPOSE mouse specific horseradish peroxidase/3-3-diamino-benzidine chromogen solution (HRP/DAB) detection immunohistochemistry kit (ab80436, Abcam, Cambridge, Massachusetts, USA). Fixed cells were observed with an inverted microscope (*Nikon, Eclipse, TE200*-U). Images captured with an infinity 2-1R CCD camera (Lumenera Corp., Ottawa, Ontario, Canada).

**Figure 7.**
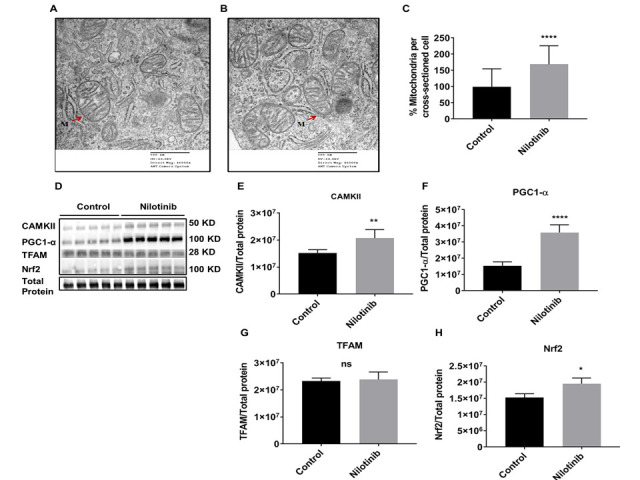
Nilotinib increased the number of mitochondria and mitochondrial biogenesis (CAMKII, PGC1-α, and Nrf2) in 3xTg-AD astroglia. The numbers of mitochondria in 3xTg astroglia were compared in the presence and absence of nilotinib (100 nM). Representative TEM image A showing the cell body of 3xTg astroglia. Representative TEM image B showing the cell body of the nilotinib-treated 3xTg-AD astroglia. (C) The number of mitochondria per cross-sectioned cell was counted (20 cells per group counted by TEM). Results are expressed as mean ± SD of n=20 per group (****P ≤ 0.0001) analyzed by unpaired Student's t-test. M: mitochondria; Scale bars: 500 μm; Magnification: 46000x. (D-H) Western blot experiments demonstrating relative levels of CAMKII, PGC1-α, TFAM, and Nrf2 in 3xTg-AD astroglia in the presence and absence of 24 hrs. 100 nM nilotinib treatment. Relative quantification for protein levels of CAMKII, PGC1-α, TFAM, and Nrf2 normalized to total protein. Results are expressed as mean ± SD of n = 5 per group (* = p < 0.05 or ** = p < 0.01 or **** = p < 0.0001) analyzed by unpaired Student's t-test.

### Immunofluorescence analysis

Immunofluorescence was utilized to detect translocation of NF-κB subunits (p50, p65, p75, also called RelA, RelB, and c-Rel, respectively) in 3xTg astroglia. Briefly, astroglia were rehydrated with TBS-T, for five minutes. Following rehydration, cells were fixed with 4% paraformaldehyde in PBS (pH 7.4) for 10 min at RT. Following the fixation procedure, antigen retrieval was performed for 30 minutes in preheated (80 °C) citrate buffer. All cells were then washed with TBS-T (three times; 5 minutes each). Cells were incubated with blocking buffer (1% bovine serum albumin (BSA) in T-BST) for 1 hour at RT. Cells were then incubated with primary antibodies: NF-κB p50 ab32360, Abcam, Cambridge, UK; 1:100; NF-κB p65: ab16502, Abcam Cambridge, UK; 1:100; and NF-κB p75: 108299, Abcam, Cambridge, UK; 1:100; anti-GFAP antibody (ab16997, abcam Cambridge, Massachusetts, USA; 1:100 dilution) to detect astroglia or Ionized calcium binding adaptor molecule 1 (Iba-1) (019-19741, wako, Saitama, Japan, 1:100 dilution) to detect microglia overnight at 4 °C. Further, cells were washed three times with TBS-T and incubated for 2 h at RT with an Alexa fluor^TM^ 488 secondary antibody (1:500; Invitrogen, Carlsbad, CA). Further, cells were washed three times with TBS-T and were mounted using mounting medium with 4′,6-diamidino-2-phenylindole (DAPI). Positively stained cells were captured on a Carl Zeiss Axioscope-2 fluorescence microscope equipped with AxioVision3 software using 20X objective. The volume density of NF-κB subunits was quantified using ImageJ software (http://rsbweb.nih.gov/ij/).

**Figure 8.**
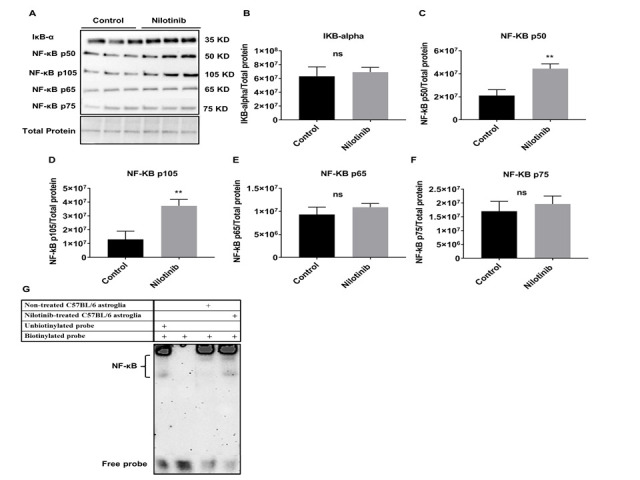
Nilotinib significantly increased the activation of NF-κB and expression of NF-κB p50/p105 subunits in C57BL/6-WT astroglia. (A) Western blot experiments demonstrating relative levels of NF-κB subunits (p50, p105, p65, and p75), and IκB-α in cultured cortical astroglia derived from C57BL/6 in the presence and absence of 24 hrs. 100 nM nilotinib treatment. (B-F) Relative quantification for protein levels of NF-κB subunits (p50, p105, p65, and p75) and IκB-α normalized to total protein. (G) Nuclear extract derived from nilotinib-treated and non-treated C57BL/6-WT astroglia were assayed for NF-κB activation by EMSA using a biotin-labeled oligonucleotide encompassing the NF-κB consensus motif. Results are expressed as mean ± SD of n = 6 per group (**P ≤ 0.01) analyzed by unpaired Student's t-test.

**Figure 9.**
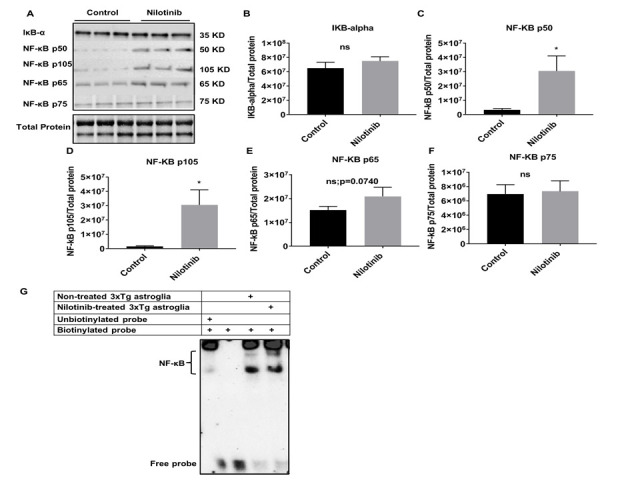
Nilotinib significantly increased the expression of NF-κB p50/p105 subunits and activation of NF-κB in 3xTg-AD astroglia. (A) Western blot experiments demonstrating relative levels of NF-κB subunits (p50, p105, p65, and p75), and IκB-α in cultured cortical astroglia derived from 3xTg in the presence and absence of 24 hrs.100 nM nilotinib treatment. (B-F) Relative quantification for protein levels of NF-κB subunits (p50, p105, p65, and p75) and IκB-α normalized to total protein. (G) Nuclear extract derived from nilotinibtreated and non-treated 3xTg-AD astroglia were assayed for NF-κB activation by EMSA using a biotinlabeled oligonucleotide encompassing the NF-κB consensus motif. Results are expressed as mean ± SD of n = 6 per group (*P ≤ 0.05) analyzed by unpaired Student's t-test.

### Transmission Electron Microscopy (TEM)

Transmission electron microscopy (TEM) was used to study the fine structure of the mitochondria and to count the number of mitochondria, as described previously [60]. Briefly, astroglia derived from 3xTg mice were treated with/without nilotinib (100 nM) for 24 hours. After incubation, cells were washed with PBS and fixed 3 hours in 3% v/v glutaraldehyde in 0.1 M Sorensen’s buffer. Cells were again fixed in 1% osmium tetroxide in 0.1 M Sorensen’s phosphate buffer for two hours, and dehydrated in a 30% to 100% ethanol series and embedded with Epon resin and infiltrated for 4 days. Later, sections were polymerized at 60 °C for 30 hours; Images were collected with Morgagni 268D electron microscope (Philips, Netherlands).

### Statistical analysis

Data was analyzed using two-tailed Student's t-tests (GraphPad Prism 6, GraphPad Software). A difference between or among groups was determined as statistically significant, if *P ≤ 0.05, **P ≤ 0.01, ***P ≤ 0.001, ****P ≤ 0.0001.

## RESULTS

### Comparing the cellular bioenergetics profile between C57BL/6 and 3xTg astroglia

The mitochondrial oxygen consumption rate (OCR) was measured in astroglia derived from cortical brain regions from control (C57BL/6) and AD (3xTg) mice (Fig. 1A). At baseline levels, OCR in 3xTg astroglia was significantly decreased compared to control groups (Fig. 1B). Similarly, OCR associated with maximal respiration capacity was significantly decreased in 3xTg astroglia *vs.* controls (Fig. 1C). Consistent with changes in parameters associated with basal and maximal respiration, spare respiratory capacity was significantly lower in 3xTg astroglia *vs*. controls (Fig. 1D). However, coupling efficiency was not significantly different in wells containing 3xTg astroglia and control cells (Fig. 1E).

### Effects of nilotinib in mitochondrial bioenergetics in C57BL/6 and 3xTg astroglia

To assess the effects of nilotinib on mitochondrial respiration, astroglia derived from C57BL/6 mice were cultured and treated with increasing concentrations of nilotinib (0, 1, 10, 100, and 1000 nM) for 24 hours. Bioenergetic parameters of basal, maximal respiration, spare respiratory capacity, and coupling efficiency were not altered in C57BL/6 astroglia as a result of nilotinib treatment (Fig. 2A-E). To further determine if the OCR rates were reflected by similar alterations in cellular energy production, total ATP production was measured. ATP levels were not altered in C57BL/6 astroglia that were treated with or without nilotinib (Fig. 2F).

**Figure 10.**
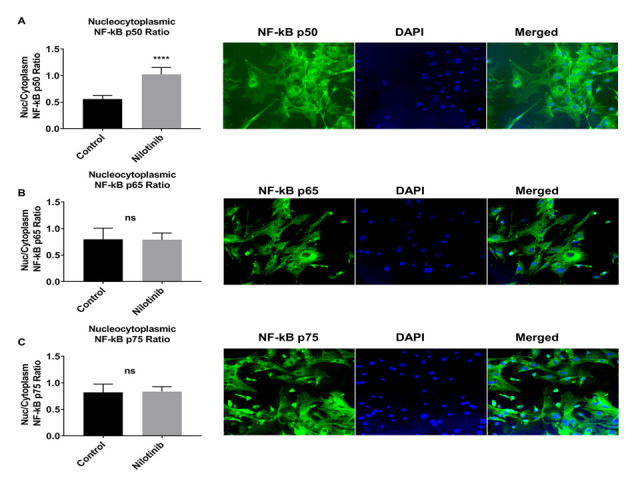
Nilotinib translocated the NF-kB p50 subunit into the nucleus of 3xTg-AD astroglia. Quantitative immunofluorescent nuclear/cytoplasmic ratios of NF-κB subunits (p50 (A), p65 (B), and p75 (C)) in 3xTg-AD astroglia. DAPI (blue) marks the nucleus. Images were captured at 100x magnification. Volume density of NF-kB subunits immunofluorescence was quantified using ImageJ software (****P ≤ 0.0001); n=5 per group analyzed by unpaired Student's t-test.

**Figure 11.**
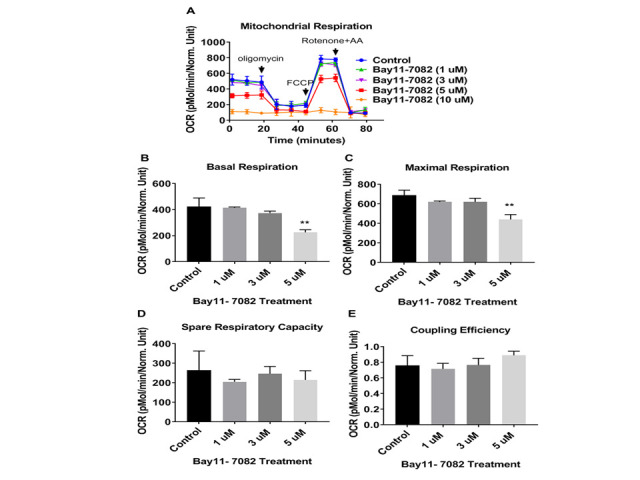
Bay11-7082 decreased mitochondrial respiration rates in 3xT-AD astroglia. (A) Kinetics graph indicating real-time OCR at baseline and after addition of oligomycin, FCCP, and rotenone/antimycin. The OCR was measured in 3xTg-AD astroglia utilizing the XF24 analyzer after 24 hours treatment with Bay11- 7082 (1, 3, 5, and 10 uM). (B) Basal respiration, (C) maximal respiration and (D) spare respiratory capacity, and (E) coupling efficiency were calculated compared between control and treated cells. Data are mean ± SD of n = 5 group (** = p < 0.01) analyzed by unpaired Student's t-test.

To assess the effect of nilotinib in the cellular bioenergetics profile of AD, astroglia derived from 3xTg mice were cultured and treated with increasing concentrations of nilotinib (0, 1, 10, 100, and 1000 nM) for 24 hours. Unlike C57BL/6 astroglia, mitochondrial OCR in 3xTg astroglia was enhanced significantly at 100 nM of nilotinib after 24 hours (Fig. 3A). At baseline, OCR in 3xTg astroglia in the presence of 100 nM nilotinib was significantly increased as compared to non-treated cells (Fig. 3B). Additionally, bioenergetics parameters of maximal respiration rates as well as spare respiratory capacity were significantly increased 24 hours after nilotinib treatment (Fig. 3C-D). However, nilotinib did not affect the coupling efficiency (coupled respiration relative to basal levels) (Fig. 3E). To confirm OCR data, total ATP production was measured in 3xTg astroglia. ATP levels were significantly increased in nilotinib-treated 3xTg astroglia (Fig. 3F).

To further determine whether c-Abl levels are altered in response to the c-Abl inhibitor, nilotinib, 3xTg and control astroglia were treated with 100 nM nilotinib for 24 hours (Fig. 2G and 3G). Nilotinib did not lower total c-Abl levels in control or 3xTg astroglia (Fig. 2H and 3H). However, nilotinib (100 nM) significantly decreased the levels of phosphorylated c-Abl in 3xTg astroglia, but not control astroglia (Fig. 2I and 3I).

### Effects of nilotinib in mitochondrial-associated proteins in C57BL/6 and 3xTg astroglia

Similar to the OCR findings in C57BL/6 astroglia, Western blot data revealed that the expression levels of key mitochondrial subunit proteins (Complexes I-V) were not altered with nilotinib treatment (Fig. 4A-B). Additionally, activity of COX and CS in the nilotinib-treated group was not altered compared to that of controls (Fig. 4C).

**Figure 12.**
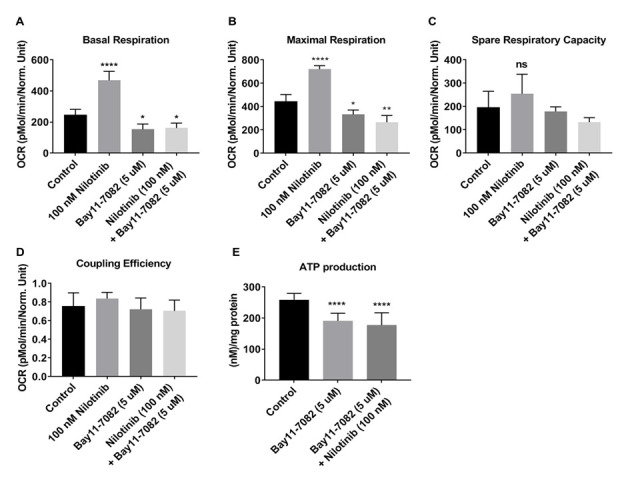
Nilotinib-induced enhancements in energy metabolism appear NF-kB-dependent, as demonstrated in 3xTg-AD astroglia. The OCR was measured in 3xTg-AD astroglia utilizing the XF24 analyzer after 24 hours treatment with nilotinib (100 nM) or Bay11-7082 (5 uM) or co-treatment with nilotinib (100 nM) + Bay11- 7082 (5 uM). (A) Basal respiration, (B) maximal respiration, (C) spare respiratory capacity, (D) coupling efficiency, and (E) ATP level were calculated compared between control and treated cells. Data are mean ± SD of n= 5 per group (* = p < 0.05 or ** = p < 0.01 or *** = p < 0.001 or **** = p < 0.0001) analyzed by unpaired Student's t-test.

Consistent with the mitochondrial OCR findings in 3xTg astroglia, Western blot results revealed a significant increase in the protein levels of NADH dehydrogenase beta sub-complex subunit 8 of Complex I, cytochrome b-c1 subunit 2 of Complex III, cytochrome c oxidase subunit 1 of Complex IV, as well as ATP synthase subunit alpha of Complex V in nilotinib-treated 3xTg astroglia *vs*. non-treated astroglia (Fig. 5A-B). The activity of CS was not altered as a function of nilotinib (Suppmentary Fig. 2). The activity of COX in the nilotinib-treated group was significantly increased compared to that of controls (Fig. 5C). However, the protein levels of Succinate dehydrogenase subunit B of Complex II were not found to be altered in nilotinib-treated 3xTg astroglia compared with non-treated ones (Fig. 5A-B).

To measure the effect of nilotinib in mitochondrial dynamics, the level of dynamin-related protein 1 (Drp1) and mitofusin 1 (Mfn1) proteins were measured by immunoblotting. Levels of Mfn1 and Drp1 were not significantly different as a function of nilotinib treatment in C57BL/6 astroglia (Fig. 6A-C), indicating that nilotinib did not induce changes in mitochondrial dynamics’ proteins in C57BL/6 astroglia. As well, nilotinib did not alter the level of Drp1 from 3xTg astroglia; however, nilotinib significantly increased the level of Mfn1 in 3xTg astroglia, indicating that nilotinib induces changes in mitochondrial mass (Fig. 6D-E). To further clarify the role of nilotinib on mitochondrial dynamics in 3xTg astroglia, we compared the number of mitochondria in nilotinib-treated *vs*. non-treated cells utilizing TEM. As shown in Fig. 7A-C, nilotinib significantly increased the number of mitochondria in 3xTg astroglia as compared to that of the non-treated group. To support the TEM data, we measured the effect of nilotinib in key regulators of mitochondrial biogenesis, including PGC-1α, CaMKII, Nrf2, and TFAM. The expression of PGC-1α, CaMKII, and Nrf2 in the nilotinib-treated 3xTg astroglia was significantly increased compared to that of controls (Fig. 7D-H). However, the protein level of TFAM was not found to be altered in nilotinib-treated 3xTg astroglia compared with non-treated cells (Fig. 7G).

### Effects of nilotinib in neuroinflammatory-associated proteins in C57BL/6 and 3xTg astroglia

To further investigate the molecular mechanisms underpinning the enhanced mitochondrial function with nilotinib, expression of various neuroinflammatory-associated proteins in C57BL/6 and 3xTg astroglia were semi-quantified with immunoblotting (Fig. 8A-F and Fig. 9A-F). Nilotinib significantly increased expression levels of NF-κB p50 and NF-κB p105 in C57BL/6 astroglia (Fig. 8C-E). However, the expression levels of IκB-α and NF-κB p75 in C57BL/6 astroglia were unaffected (Fig. 8B and 8F). Further, EMSA was used to verify the effect of nilotinib on NF-κB activation and binding. Nilotinib increased transcriptional activity of NF-κB in C57BL/6 astroglia (Fig. 8G).

In addition, nilotinib increased expression levels of NF-κB p50 and NF-κB p105 subunits in 3xTg astroglia (Fig. 9C-D). In contrast, the expression levels of IκB-α, NF-κB p65, and NF-κB p75 subunits in 3xTg astroglia were unaffected (Fig. 9B, 9E, and 9F). Further, EMSA data verify that nilotinib significantly increased transcriptional activity of NF-κB in 3xTg astroglia (Fig. 9G).

Stimulation with specific inducers activate NF-κB signaling pathways resulting in the translocation of NF-κB dimers to the nucleus to activate NF-κB target genes [71]. In the current study, immunofluorescence (IF) was utilized to verify the effect of nilotinib on NF-κB translocation. IF data in 3xTg astroglia indicated that nilotinib (100 nM) treatment significantly translocated NF-κB p50 to the nucleus but had no effect on the translocation of NF-κB p65 or NF-κB p75 subunits (Fig. 10A-C).

### Effects of nilotinib and NF-κB inhibitor, Bay11-7082, on mitochondrial respiration in 3xTg astroglia

To investigate whether nilotinib increased mitochondrial respiration in 3xTg astroglia through the NF-κB signaling pathway, astroglia were treated with either a NF-κB inhibitor (Bay11-7082 (1, 3, 5, and 10 µM)) alone, or co-treated with nilotinib (100 nM) for 24 hours. The mitochondrial OCR in 3xTg astroglia was decreased significantly at 5 µM of Bay11-7082 after 24 hours (Fig. 11A). At basal and maximal level, OCR in 3xTg astroglia in the presence of Bay11-7082 (5 µM) was significantly decreased as compared to non-treated cells (Fig. 11B-C). Additionally, bioenergetics’ parameters of spare respiratory capacity, as well as spare coupling efficiency, were unchanged after Bay11-7082 treatment (Fig. 11D-E). Although Bay11-7082 decreased OCR level in 3xTg astroglia, co-treatment of Bay11-7082 (5 µM) and nilotinib (100 nM) did not compensate mitochondrial deficits (Fig. 12A-C). Spare respiratory and coupling efficiency were not affected with co-treatment of Bay11-7082 (5 uM) and nilotinib (100 nM) (Fig. 12D-E). To further determine if the mitochondrial respiration rates were reflected by similar alterations in cellular energy production, total ATP production was measured. ATP levels were significantly decreased in 3xTg astroglia that were treated with nilotinib or co-treated with Bay11-7082 (Fig. 12F).

## DISCUSSION

We report, for the first time, the effects of nilotinib treatment on brain astroglia derived from the cortex of 3xTg-AD versus C57BL/6-WT mice. The results showed cortical astroglia from 3xTg mice exhibited *significant* declines in parameters of mitochondrial bioenergetics as compared to the control group. This study is the *first* to demonstrate increased mitochondrial function of 3xTg astroglia as a result of nilotinib treatment (100 nM). Also, for the first time we provide evidence that nilotinib’s benefit in mitochondria involves an NF-κB-dependent pathway. Nilotinib has been previously shown to induce apoptosis in cancer cells at micromolar rather than nanomolar concentrations [12, 72-75]. However, data from our study indicate that nilotinib is effective in 3xTg astroglia at nanomolar concentrations. Growing evidence suggests that mitochondrial dysfunction contributes to early events in the pathogenesis of AD [7, 40, 76, 77]. Therefore, the current study has important implications for the treatment of disorders affected by dysfunctional mitochondria, including AD.

**Figure 13.**
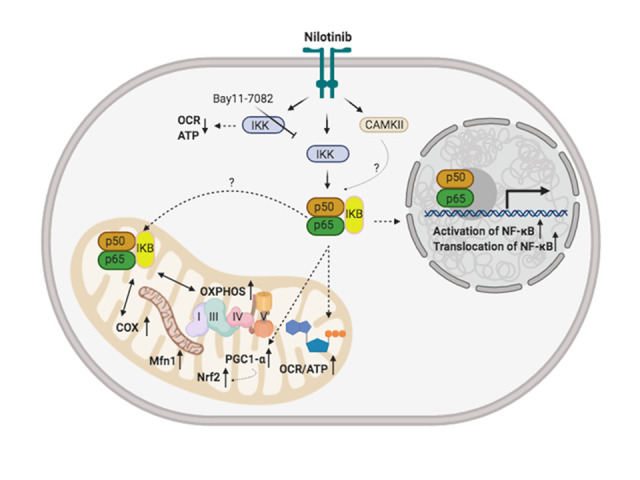
This putative pathway suggests that nilotinib improves mitochondrial function through nuclear factor κ B (NF-κB)-dependent signaling in 3xTg-AD astroglia. It is hypothesized that nilotinib activates CAMKII as well as a signal cascade resulting in degradation of the NF-κB subunit IκB. The NF-κB dimer then translocates from the cytoplasm to the nucleus where it binds to a DNA consensus sequence of target genes. It is also hypothesized that nilotinib triggers the NF-κB complex (downstream target of CAMKII) to move into the mitochondrion, where is thought to interact with OXPHOS genes that leads to bioenergetic improvement (ATP, oxygen consumption rate (OCR)), as well as an increase in the expression of mitochondrial complex proteins subunits (Complexes I, III, IV, and V), cytochrome c oxidase (COX), mitochondrial biogenesis (PGC1-α and Nrf2), and mitochondrial dynamics (Mfn1). Findings from our current study importantly show that mitochondrial OCR and ATP levels were significantly reduced in the presence of the IKK inhibitor (Bay11-7082). This figure was developed using the BioRender online software tool.

Different types of brain cells have unique features of mitochondrial function, and display explicit roles related to their metabolic function. A previous proteomic study designates that the mitochondrial function and morphology is various in different types of brain cells [78]. A better understanding of mitochondrial impairment in AD requires a cell-type specific approach. Although astroglia and neurons are linked in their energy metabolism, they are still different types of brain cell that utilize a very different metabolic profile. Neuronal cells, during periods of enhanced activity, produce the toxic fatty acids (FA) that are transported into astroglia, which can be detoxified in mitochondria instead of being dealt with in neurons [79]. Unlike astroglia, neuronal cells have a restricted capacity to up-regulate glycolysis or to counteract oxidative damage [80, 81]. Additionally, neurons rely on OXPHOS to meet their energy requirements; therefore, mitochondrial dysfunction leads to increased generation of reactive oxygen species (ROS), energy failure, and ultimately neuronal cell death. Under environmental and cellular stress conditions, astroglia have their distinct metabolic reprogramming mediated via mitochondrial dynamics, which determine the inflammatory features of glia [82, 83]. Other studies show that metabolic activity in astroglia may actually increase with age [84]. Increased metabolic activity in astroglia was linked with an age-dependent increase in inflammatory pathways, including NF-κB signaling [85, 86]. Further, inflammatory cytokines in astroglia triggered OXPHOS as well as mitochondrial biogenesis [84]. Human astroglia infected with human immunodeficiency virus (HIV) displayed mitochondrial damage, severe inflammation, and ultimately cell death. On the other hand, HIV-infected astroglia modulates inflammatory responses and regulation of mitochondrial dynamics followed by mitophagy [87]. These opposed scenarios with regard to modulation of mitochondrial dynamics regulate cell viability. Investigators have speculated that mitophagy in HIV-infected astroglia may reduce inflammation via removing damaged mitochondria that leads to cell viability [60, 87]. Interestingly, Lonskaya et al. show that nilotinib improves autophagic machinery, leading to Aβ clearance in transgenic amyloid precursor protein (APP) mice, thus suggesting therapeutic benefits for AD [88]. A current study also shows that mitochondrial OCR was significantly lower in AD astroglia as compared to control and nilotinib increased mitochondrial function and inflammatory responses. It has also been suggested that the astroglia response to nilotinib leads to increased inflammatory responses that support the functionality switch of astroglia to increase metabolic activity for energy support. Also, nilotinib increases the expression of mitochondrial dynamics, which might act as a compensatory mechanism to preserve energy by balancing proteins from one healthy mitochondrion to another mitochondrion that might be damaged.

The cumulative evidence has reported that mitochondrial dysfunction plays an important role in neurodegenerative disorders including AD. The present study demonstrates that nilotinib regulates brain bioenergetics in 3xTg astroglia. Our data show that nilotinib treatment of 3xTg astroglia increased the mitochondrial respiration rate, ATP levels, and the expression of mitochondrial complex subunit proteins. Metabolic investigations into synaptic mitochondia show that a ~25% reduction in Complex I activity and >70% reduction in Complexes III and IV activity results in depletion of ATP and OCR levels [89, 90]. Therefore, a significant portion of oxidative phosphorylation in synaptic mitochondria is regulated via the activity of Complex I and Complexes (III and IV), respectively. In the current study, nilotinib increased the expression of mitochondrial subunits (Complexes I and III-V) that may lead to increased newly-generated synapses.

A decrease in mitochondrial respiration rate in 3xTg cells is also consistent with literature indicating mitochondrial function impairment in 3xTg mouse models [91, 92]. Previously, our laboratory identified significant decreases in brain glucose uptake rates and decreases in the activity of the terminal mitochondrial enzyme (COX or Complex IV) in 3xTg cortex [8]. In the present study, nilotinib increased the activity of COX (Complex IV) in 3xTg astroglia. These results suggest that nilotinib improves the function of respiratory complexes in 3xTg astroglia. However, nilotinib had no effect on protein levels of mitochondrial complex subunits in control astroglia. Together, these data indicate that nilotinib improves mitochondrial function under pathological states.

Additionally, increase in mitochondrial oxidative metabolism is paralleled by increase in the level of COX suggesting an enhanced mitochondrial biogenesis [93]. Moreover, several reports link mitochondrial bioenergetics to mitochondrial dynamics [94-96]. Additionally, impaired mitochondrial function is consistent with altered mitochondrial dynamics in AD [49, 77, 97-101]. Some studies report increased levels of mitochondrial fission protein Drp1 and decreased levels of mitochondrial fusion protein Mfn2 in AD brain [102, 103], which lead to loss of respiratory capacity, mitochondrial damage and thus a decreased energy metabolism [5, 104, 105]. Other studies report that mitochondrial dynamic inhibitors reduced mitochondrial metabolism as well as the activity of the ETC, which leads to neurodegeneration in AD [100, 105-108]. The Mfn-mediated fusion is an important marker to maintain proper ETC function and maintain OXPHOS. In fact, a deficiency in mitochondrial fusion has been revealed to result in mitochondrial dysfunction [109]. For example, double knock out Mfn1/2 mice show reduced number of mtDNA, OCR, and complex II activity [110]. Our current study reports that nilotinib increased the level of mitochondrial fusion marker (Mfn1), which would ascribe specific features to mitochondria for various cellular metabolic procedures, such as up regulation of mitochondrial bioenergetics (OCR, ATP) and OXPHOS.

Mitochondrial fusion has been proposed to provide a new strategy for treating mitochondria-related disorders, such as neurodegenerative disorders [111]. Even mutant-DNA mitochondria can fuse with healthy mitochondria of the same cell, which allows WT-DNA mitochondria to compensate for defects in mutant-DNA mitochondria by sharing RNA and/or protein components [112, 113]. Additionally, mitochondrial fusion can rescue two mutant mitochondria by cross-complementation to one another [114]. It is also suggested that mitochondrial fusion functions as a protective response against stress conditions, carried out by the main participants in the mitochondrial fusion. Disruption in mitochondrial fusion markers (Mfn1/Mfn2) caused severe reduction of mitochondrial DNA (mtDNA) levels [110]. Additionally, mitochondrial fusion disorder significantly decreases mitochondrial function and ultimately causes death in a mouse model with high levels of mtDNA mutations, proposing a protective role for Mfn1/2 against mtDNA mutations [110]. Overall, mitochondrial fusion can lead to compensation of damage, thus rescuing damaged organelles and failure of mitochondrial fusion may lead to neurodegenerative diseases. The current study shows that nilotinib increased the expression of mitochondrial fusion. It is suggested that mitochondrial fusion acts as a compensatory mechanism to preserve energy by balancing proteins from one healthy mitochondrion to another, which might be damaged. Therefore, mitochondrial fusion may rescue damaged organelles and create a pool of healthy mitochondria by increasing fusion regularity without compromising the mitophagy of dysfunctional mitochondria [115]. The current study suggests that Mfn1 is compensating for an increase in mitochondrial biogenesis. These findings propose that regulation of mitochondrial dynamics and biogenesis signaling may indicate a new mitochondria-targeted therapeutic strategy for AD.

Emerging evidence has also revealed that mitochondrial dysfunction triggers inflammation [116]. Among a number of different transcription factors that are involved in the inflammation NF-κB is a key inflammatory regulator with activation in glia following brain injury or infection [117-119]. Several studies reported that NF-κB controls inflammation in astroglia [120-122]. Additionally, the activity of NF-κB has been reported in glia isolated from AD rodent models, a Huntington’s disease mouse model, and the spinal cord of patients with amyotrophic lateral sclerosis (ALS) [123-125]. Also, glial NF-κB is associated with the pathology of neurodegenerative disorders such as AD [126]. The NF-κB pathway was activated in glia cultures in response to Aβ treatment, which leads to increased expression of inflammatory cytokines such as interleukin-6 and interleukin-1 beta, and tumor necrosis factor α (TNFα) [84, 127-129]. Jiang and Cadenas 2014 found that increased inflammation triggers mitochondrial OXPHOS in astroglia [84]. These findings are consistent with our study, while nilotinib increases the expression of inflammatory marker (NF-κB) and OXPHOS. These data suggest NF-κB might play a compensatory role and can have beneficial effects in preserving brain health through an immune response. It is also important to consider NF-κB is not only involved in inflammation, but also plays a critical role in learning and memory formation [130-132]. Therefore, our finding suggests that up regulation of NF-κB via nilotinib might lead to memory improvement. Further research examining the effects of nilotinib in improving memory in AD mice is required.

Both mitochondrial dysfunction and neuroinflammation play critical roles in AD progression; However, molecular mechanisms underpinning enhanced mitochondrial function with nilotinib in astroglia are lacking. NF-κB regulates energy metabolism and metabolic activity by upregulating mitochondrial respiration in cancer cells [53]. Nevertheless, how NF-κB controls mitochondrial function in AD brain cells is not understood [57]. To assess whether energy metabolism is enhanced by nilotinib through NF-κB associated pathways, 3xTg astroglia were treated with nilotinib alone or with Bay11-7082, an NF-κB inhibitor. Mitochondrial OCR levels were reduced in the presence of Bay11-7082; however, co-treatment of Bay11-7082 and nilotinib did not compensate for any mitochondrial deficits. These findings suggest that nilotinib could trigger mitochondrial function through an NF-κB-dependent signaling pathway.

Furthermore, immunofluorescence confirmed that the NF-κB p50 subunit is translocated from the cytoplasm to the nucleus in nilotinib-treated 3xTg astroglia, showing that nilotinib initiates the transcriptional induction of NF-κB-inflammatory signaling. Moreover, immunoblotting and EMSA demonstrated that nilotinib increased transcriptional activity of NF-κB in 3xTg astroglia, which might be explained by increased NF-κB nuclear translocation under nilotinib treatment. These findings indicate that the mechanism underlying the effect of nilotinib on cellular respiration involves an NF-κB-dependent pathway.

Several studies show that NF-κB has been specially linked to CaMKII activation [133, 134]. CaMKII plays critical roles in regulating inflammation as well as various mitochondrial functions, such as mitochondrial biogenesis [92, 135]. An increase in CaMKII expression, in the presence of increased NF-κB transcriptional activity, as found here with nilotinib in 3xTg astroglia, is also consistent with literature indicating that CaMKII (upstream target) is required for NF-κB activation [136]. PGC-1α, a downstream target of CaMKII that has been implicated in mitochondrial biogenesis, was significantly increased as a function of nilotinib. The PGC-1α is involved in AD pathogenesis and upregulation of PGC-1α improves mitochondrial biogenesis through induction of transcription factor Nrf2, which is linked to NF-κB in pathological conditions [137-139]. The current study found upregulation of PGC-1α, in nilotinib-treated 3xTg astroglia, consistent with increased Nrf2 (downstream target of PGC-1α), which is a key modulator of mitochondrial metabolism and biogenesis [140-142]. These findings are consistent with previous studies indicating a role of Nrf2 in inflammation, mitochondrial biogenesis and bioenergetics and its interaction with PGC-1α in the context of neurodegeneration [64, 143]. Overall, our study suggests that nilotinib may increase the number of mitochondria through a CaMKII- PGC-1α-Nrf2 pathway, which is influenced by NF-κB dynamics in 3xTg astroglia.

## Conclusions

Emerging evidence suggests that defects in mitochondrial function are associated with bioenergetic deficiency and neuroinflammation, and could serve as early biomarkers of AD. The present results have important implications for treatment of disorders with mitochondrial dysfunction including AD. The major finding of this study is that nilotinib treatment stimulated oxidative phosphorylation in 3xTg astroglia with upregulation of mitochondrial complex proteins subunits and COX activity. Additionally, nilotinib increases mitochondrial function and dynamics through altered CaMKII-PGC-1α-Nrf2 pathway activity that are involved in the development of neurodegeneration. Nilotinib also increased mitochondrial function via the NF-κB signaling pathway. Collectively, these results contribute to our understanding of the effects of nilotinib on regulating brain mitochondrial bioenergetics and biogenesis (Fig. 13).

## Supplementary Materials

The Supplemenantry data can be found online at: www.aginganddisease.org/EN/10.14336/AD.2020.0910.


