# Supplementary material for: Nilotinib Improves Bioenergetic Profiling in Brain Astroglia in the 3xTg Mouse Model of Alzheimer’s Disease
Source: Aging Dis. 2021 Apr 1;12(2):441–65. doi: 10.14336/AD.2020.0910 (PMC7990369; doi:10.14336/AD.2020.0910)
Supplement: Supplementary file 1 [file AD-12-2-441-s.pdf]

SUPPLEMENTARY DATA

Nilotinib Improves Bioenergetic Profiling in Brain Astroglia in the 3xTg Mouse Model of Alzheimer's Disease

Aida Adlimoghaddam[1,*], Gary G. Odero[1], Gordon Glazner[1,2], R. Scott Turner[3], Benedict C. Albensi[1,2,*]

© 2020. Adlimoghaddam A. et al. Published online at http://www.aginganddisease.org/EN/10.14336/AD.2020.0910

SUPPLEMENTARY DATA

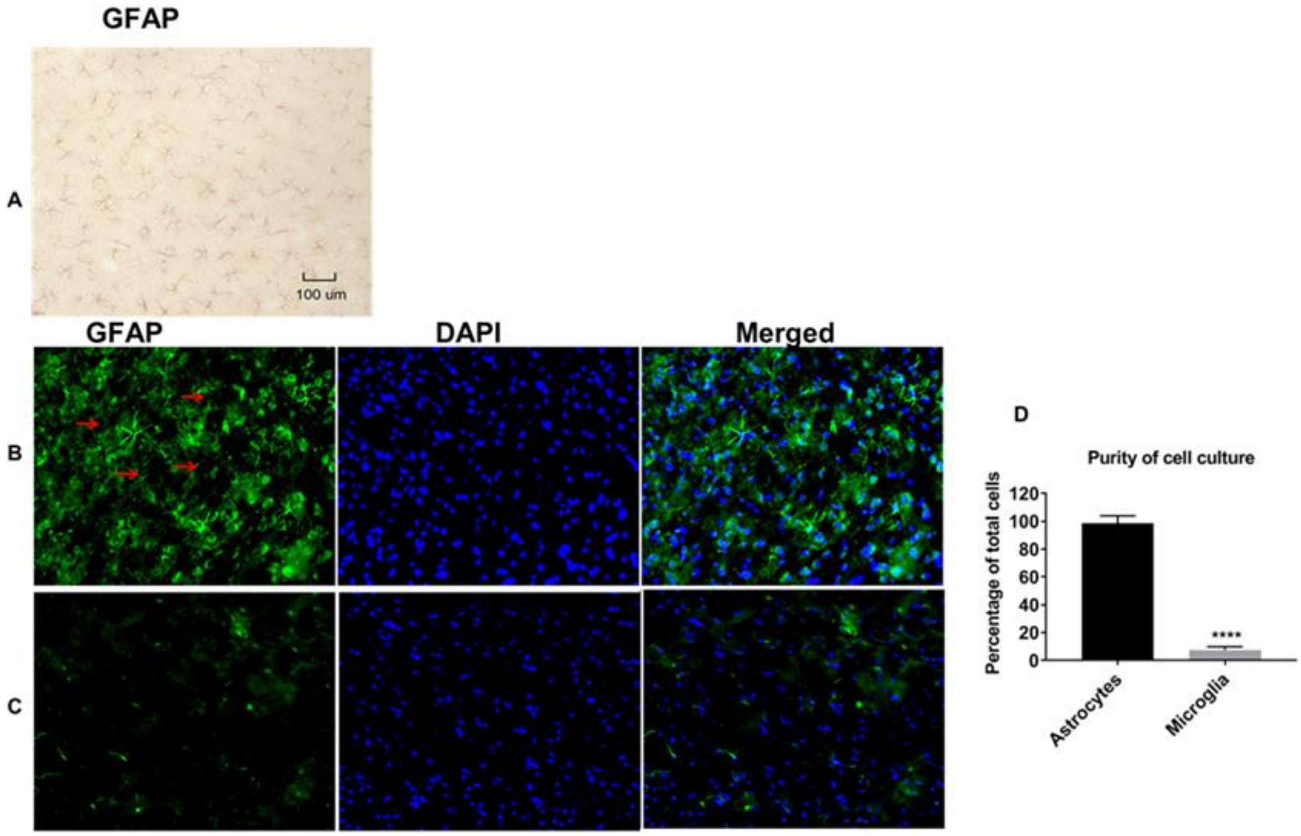

Supplementary Figure 1. Characterization of culture purity. Both immunohistochemistry **(A)** and immunofluorescence **(B)** were utilized to detect astrocytes. Purity of cell culture was determined via immunofluorescence using markers specific for detecting astrocytes (GFAP) and microglia (Iba-1). Red arrows indicate the astrocytes. Nuclei were labeled blue with DAPI. The percentages of astrocytes and microglia were determined. Results are expressed as mean ± SD of n = 5 (**** = $p < 0.0001$).

© 2020. Adlimoghaddam A. et al. Published online at http://www.aginganddisease.org/EN/10.14336/AD.2020.0910

SUPPLEMENTARY DATA

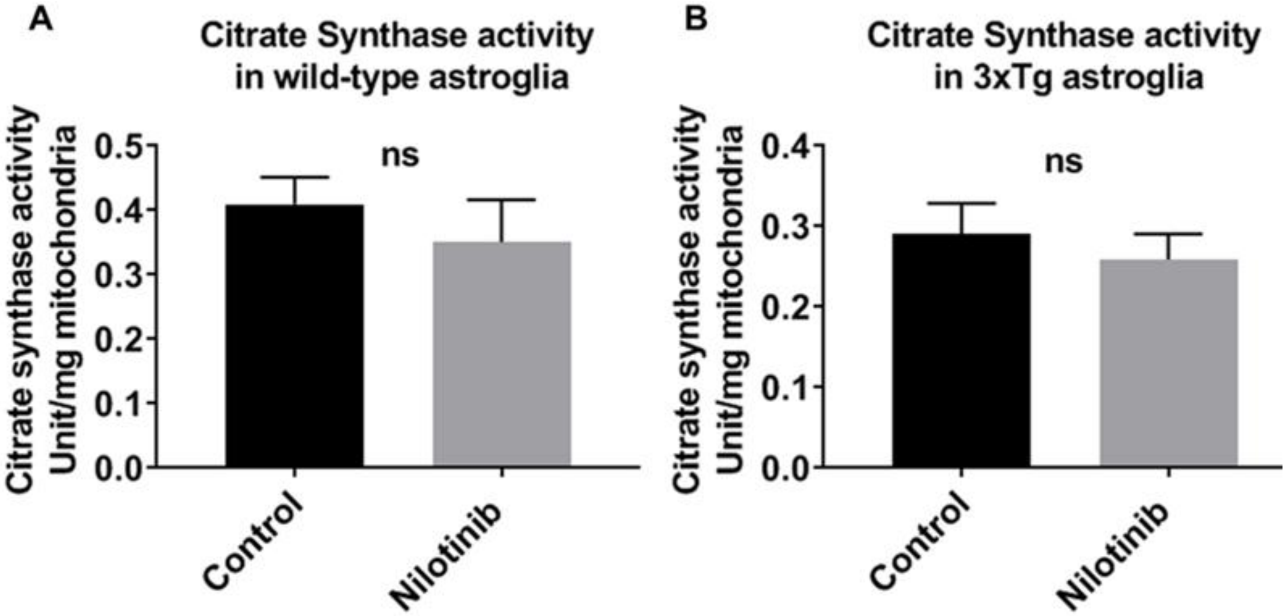

Supplementary Figure 2. Nilotinib did not alter the activity of citrate synthase (n = 6/group) in C57BL/6-WT and 3xTg-AD astroglia. Specific enzyme activity of citrate synthase was measured in C57BL/6-WT **(A)** and 3xTg-AD **(B)** astroglia in presence and absence of 100 nM nilotinib treatment. Results are expressed as mean ± SD of n = 6 per group (*$P \leq 0.05$) analyzed by unpaired Student's t-test.

© 2020. Adlimoghaddam A. et al. Published online at http://www.aginganddisease.org/EN/10.14336/AD.2020.0910
